# Author Correction: Regulation of synaptic architecture and synaptic vesicle pools by Nervous wreck at *Drosophila* Type 1b glutamatergic synapses

**DOI:** 10.1038/s12276-018-0132-z

**Published:** 2018-08-20

**Authors:** Joon Haeng Hur, Sang-Hee Lee, A-Young Kim, Young Ho Koh

**Affiliations:** 1 ILSONG Institute of Life Science, Hallym University, Anyang, Republic of Korea; 2 Department of Bio-Medical Gerontology, Hallym University Graduate School, Chuncheon, Republic of Korea; 3 BioMedical Research Center, Korea Advanced Institute of Science and Technology, Daejeon, Republic of Korea

**Correction to**: *Experimental & Molecular Medicine* **50**:e642; 10.1038/emm.2017.303; published online: 23 March 2018

After online publication of this article, the authors noticed an error in the “Acknowledgements” section.

The correct statement of this article should have read as below.

ACKNOWLEDGEMENTS

The Korea Basic Science Institute kindly provided HVEM and IMOD analysis. This work was carried out with the support of the Cooperative Research Program for Agriculture Science & Technology Development (No.: PJ01332403), the Rural Development Administration and the Basic Science Research Program through the National Research Foundation of Korea (NRF) funded by the Ministry of Education, Science and Technology (no. 2015R1D1A3A01018497).

The authors apologize for any inconvenience caused.

